# Comparative analysis of breast-conserving surgery versus reconstruction post-mastectomy: a BREAST-Q™ assessment

**DOI:** 10.3332/ecancer.2026.2095

**Published:** 2026-03-18

**Authors:** Kristian Bugeja, Alexia Mercieca, Kristina Attard, Serkan Ilgun, John Agius

**Affiliations:** 1 Mater Dei Hospital, MSD2090 Msida, Malta

**Keywords:** breast-conserving surgery, breast reconstruction, BREAST-Q, patient-reported outcomes, quality of life, mastectomy

## Abstract

**Objective:**

To compare patient-reported outcomes of breast-conserving surgery (BCS) and breast reconstruction post-mastectomy (BRPM) at Mater Dei Hospital using the BREAST-Q™ questionnaire, with a focus on multiple quality-of-life domains including satisfaction with breasts, psychosocial well-being, physical well-being, sexual well-being, fatigue, cancer worry and impact on work.

**Methods:**

This retrospective audit analysed 46 patients treated between 2017 and 2020 at Mater Dei Hospital, Malta: 23 underwent BCS via wide local excision, and 23 underwent two-stage implant-based reconstruction following mastectomy. To reduce variability, only cases performed by a single breast surgeon were included, and age distributions were matched between groups. Patient-reported outcomes were assessed using the validated BREAST-Q™ version 1.0 questionnaire. Statistical comparisons between the BCS and BRPM groups were conducted using the Mann-Whitney U test.

**Results:**

BCS patients reported higher scores in physical well-being (70.0% versus 64.6%, p = 0.01) and slightly better sexual well-being (73.3% vs versus 66.7%, p = 0.83), while BRPM patients showed higher psychosocial well-being (74.0% vs versus 69.4%, p = 0.17). Satisfaction with breast outcomes appeared higher in the BCS group when expressed as a percentage of the maximum possible score (83.5% vs versus 63.3%, p = 0.43), though raw scores favoured BRPM. Cancer worry, fatigue and impact on work scores were similar between groups, with no statistically significant differences.

**Conclusion:**

Both BCS and BRPM yielded broadly comparable patient-reported outcomes across most domains. BRPM was associated with higher psychosocial well-being, while BCS patients experienced better physical and sexual well-being. The only statistically significant difference was found in physical well-being, favouring BCS. These findings underscore the importance of personalised counselling and shared decision-making to align surgical choices with patient values and expectations.

## Introduction

Breast cancer treatment options include breast-conserving surgery (BCS) and breast reconstruction post-mastectomy (BRPM), each presenting distinct clinical and psychosocial considerations. While BCS aims to preserve the breast, BRPM offers the opportunity for aesthetic restoration following mastectomy. The decision between these options often involves considering factors such as cosmetic outcomes, psychological impact and quality of life. The breast Q questionnaire has emerged as a validated tool to assess patient-reported outcomes in breast surgery, providing valuable insights into various domains of patient experience.

## Objective

This audit aimed to compare the outcomes of BCS and BRPM performed at Mater Dei Hospital, Malta, between 2017 and 2020. The primary objective was to assess patient-reported outcomes using the breast Q questionnaire, evaluating satisfaction with breasts, psychosocial well-being and physical well-being. By analysing data from both surgical groups, the audit sought to identify significant differences in outcomes, offering insights to inform patient counselling and surgical decision-making for breast cancer treatment.

## Methods

This audit involved a retrospective analysis of patient data from 2017 to 2020 at Mater Dei Hospital, Malta. It included 46 patients: 23 who underwent BCS via wide local excision and 23 who underwent two-stage reconstruction following mastectomy, with initial tissue expander placement followed by implant-based reconstruction. To minimise variability, only cases performed by a single breast surgeon were included. Age distributions were matched between groups to reduce demographic bias and enable a more accurate comparison of outcomes. Patient-reported outcomes were assessed using the validated Breast Q™ version 1.0 questionnaire, with data collected on demographics, surgical details and postoperative results. Particular focus was given to satisfaction with breasts, psychosocial well-being and physical well-being to ensure a standardised evaluation across both groups. Statistical comparisons between the BCS and BRPM groups were conducted using the Mann-Whitney *U* test.

## Results

This audit included 23 patients who underwent BCS and 23 who underwent BRPM. The ages of patients in the BCS group ranged from 30 to 60 years, with an average age of 54.6 years. In the BRPM group, patients’ ages ranged from 41 to 58 years, with an average age of 50.8 years. These ranges represent the typical demographic distribution of patients undergoing these procedures at Mater Dei Hospital. Patient-reported outcomes were assessed using the Breast Q™ questionnaire, focusing on domains such as psychosocial well-being, sexual well-being, cancer worry, fatigue, impact on work, physical well-being postoperatively and satisfaction with breast postoperatively.

Sexual well-being scores were slightly higher in the BCS group, with a mean of 22.0 (73.3%, SD: 4.5), versus 20.0 (66.7%, SD: 3.5) in the BRPM group. This difference was also not statistically significant (*p* = 0.83), suggesting broadly comparable outcomes between groups in this domain. While not statistically significant, this trend may hold clinical relevance and warrants further investigation in larger cohorts.

Cancer worry scores were similarly distributed across both groups. In this domain, a higher score indicated greater concern regarding cancer recurrence. Patients in the BCS group reported a mean score of 33.6 (84.0%, SD: 4.3), while those in the BRPM group had a mean of 32.8 (81.9%, SD: 4.2), with no significant difference observed (*p* = 0.59). Fatigue scores were consistent between the groups, whereas a higher score reflects a better outcome. The BCS group reported a mean of 34.8 (86.9%, SD: 4.5), while the BRPM group had a comparable mean of 34.5 (86.3%, SD: 4.5), with a *p*-value of 0.64.

A statistically significant difference was observed in physical well-being, with the BCS group reporting a higher mean score of 21.0 (70.0%, SD: 1.8) compared to 19.4 (64.6%, SD: 5.1) in the BRPM group (*p* = 0.01), indicating greater physical comfort postoperatively among BCS patients.

The impact on the ability to work was similar between groups. Patients in the BCS group scored a mean of 24.7 (77.3%, SD: 2.0), while the BRPM group scored 23.8 (74.3%, SD: 6.7), with no statistically significant difference (*p* = 0.75).

Satisfaction with breast outcomes showed a slight difference in raw scores. The BRPM group reported a mean of 38.0 (63.3%, SD: 11.9), while the BCS group reported 36.7 (83.5%, SD: 10.8). This discrepancy in percentages reflects the differing maximum scores used for each group (60 for BRPM and 44 for BCS). The difference between groups was not statistically significant (*p* = 0.43).

Psychosocial well-being scores were higher in the BRPM group, with a mean of 37.3 (74.0%, SD: 7.3), compared to 34.7 (69.4%, SD: 6.8) in the BCS group (*p* = 0.17). This finding contrasts with overall satisfaction trends, where BRPM patients reported lower percentage scores. The higher psychosocial scores in the BRPM group may reflect psychological reassurance associated with undergoing two-stage reconstruction, potentially supported by structured counselling during the planning process and a sense of restoration following mastectomy.

Overall, patient-reported outcomes were broadly comparable between the two surgical groups, with minor differences observed across domains. Psychosocial well-being was higher in the BRPM group, while the BCS group reported better outcomes in sexual and physical well-being. Most domains, including cancer worry, fatigue and impact on work, showed no significant differences. The only statistically significant finding was in physical well-being, favouring the BCS group.

## Discussion

This audit offers valuable insights into how patients perceive their outcomes following BCS versus BRPM, as measured by the BREAST-Q™ instrument. Both surgical techniques were associated with favourable quality-of-life scores, though they differed in specific domains. BCS was linked to better physical well-being and slightly enhanced sexual well-being, while BRPM was associated with improved psychosocial functioning. No statistically significant differences emerged in terms of fatigue, cancer-related anxiety or work-related impact. Comparable trends have been noted in previous studies, which similarly highlight unique benefits of both approaches without a universally superior option [1, 2, 5, 7].

The improved physical well-being among BCS patients aligns with earlier reports indicating that less invasive surgery may result in fewer chest wall symptoms and better postoperative comfort [1]. Since BCS generally preserves more natural tissue and avoids implant-related complications, these patients may experience less discomfort. Not all investigations have supported this advantage, with some attributing lower physical scores in BCS patients to radiotherapy side effects [4]. In our cohort, such adverse effects may have been limited or offset by the absence of extensive reconstruction. Thus, these results reinforce the idea that physical well-being tends to favour BCS when it is clinically appropriate [1, 7].

Sexual well-being was modestly higher in the BCS group. This is consistent with the broader understanding that retaining the natural breast can contribute positively to body image and intimate relationships. Breast removal and reconstruction can affect sensation and psychological perception of femininity, which may alter sexual experiences [3, 5]. BCS, by conserving anatomy and sensory function, may thus better support patients’ sexual identity. These findings are important when discussing treatment with patients who may be concerned about postoperative intimacy.

Psychosocial well-being scores were unexpectedly higher among BRPM patients. This suggests that, despite undergoing a more extensive operation, reconstruction may effectively restore emotional and social confidence. While numerous studies have praised the psychological benefits of BCS [1, 5], our data suggest that modern reconstructive methods can offer comparable or even greater support to patients' emotional recovery. These findings are consistent with prior analyses where reconstruction mitigated the negative effects of mastectomy and improved body image [4, 7]. Variability in expectations, cultural norms and the act of making a proactive choice for reconstruction may explain this trend. Further prospective studies could clarify these dynamics.

Breast satisfaction warrants particular attention. Though absolute BREAST-Q™ scores were higher in the BRPM group, BCS patients demonstrated a greater proportion of maximum satisfaction within their specific module. This highlights challenges in cross-module comparisons within the BREAST-Q™ and underscores that both cohorts reported meaningful satisfaction with outcomes. BCS patients may derive confidence from retaining their natural anatomy, while BRPM patients benefit from the aesthetic restoration offered by reconstruction. Previous work similarly reports comparable satisfaction levels between the two modalities [1, 5], with some evidence favouring BCS [3], It is essential to recognise that patient satisfaction is multifactorial and can evolve over time, influenced by expectations, age and postoperative changes [1].

Domains such as cancer worry, fatigue and occupational impact revealed no significant variation between surgical strategies. This suggests that long-term functional recovery and emotional adjustment are achievable regardless of surgical choice. The assumption that mastectomy might alleviate recurrence anxiety is not supported by our data, consistent with earlier studies, which found similar levels of cancer worry across surgical types [6]. Fatigue and work limitations likely stem more from adjuvant therapy and general health than from the surgical method. Other research supports these observations, indicating that overall functional status post-treatment is not significantly dependent on the surgical routes.

It is important to acknowledge the limitations of this audit. Being retrospective in nature, the study is susceptible to selection bias. Treatment decisions were based on tumour features and patient preferences, which may influence reported outcomes. Absence of baseline BREAST-Q™ scores precludes assessment of preoperative differences. The relatively small sample size and single-centre design may limit generalisability. Additionally, while score percentages were used to enhance comparability, the inherent differences in BREAST-Q™ modules complicate direct comparisons. Despite these caveats, our findings remain consistent with broader research, lending support to their validity.

These findings reinforce the value of tailoring surgical choices to each patient’s needs and priorities. When appropriate, BCS offers a pathway that may help preserve physical comfort and natural breast sensation, while immediate reconstruction remains an important option for individuals who place greater emphasis on psychological well-being and aesthetic restoration following mastectomy. These considerations should form part of a nuanced, patient-centred discussion during surgical planning.

## Conclusion

This analysis comparing BCS and BRPM shows that both surgical strategies produce favourable patient-reported outcomes. BCS patients experienced superior physical comfort and marginally better sexual well-being, reflecting the benefits of a more conservative, anatomically preserving approach. Conversely, those who underwent reconstruction reported stronger psychosocial recovery, indicating that reconstructive options can restore body image and emotional well-being after mastectomy. Satisfaction with breast appearance was similarly high across both cohorts when considered within each procedure’s context. There were no notable differences in fatigue, recurrence anxiety or return to work.

These results support the continued recommendation of breast-conserving options when clinically suitable. Nonetheless, when mastectomy is indicated or chosen, immediate reconstruction can effectively preserve or restore quality of life. Decision-making should be tailored to the individual, factoring in personal values, lifestyle, aesthetic expectations and psychological readiness. Overall, our findings offer reassurance that either path can lead to a satisfactory and meaningful recovery, supporting a shared decision-making model in breast cancer treatment.

## List of abbreviations

BCS, Breast-conserving surgery; BRPM, Breast reconstruction post-mastectomy; BREAST-Q™, Breast reconstruction and augmentation evaluation – quality of life questionnaire; MDH, Mater Dei Hospital; QOL, Quality of life; SD, Standard deviation.

## Conflicts of interest

We declare that there is no conflicts of interest regarding the publication of this paper.

## Funding

No funding was required for this audit.

## Ethical approval

This study received ethical approval from the Clinical Research Ethics Committee at Mater Dei Hospital, Malta. Informed consent was obtained from all participants and data were anonymised before analysis.
